# Comment on 'The distribution of antibiotic use and its association with antibiotic resistance'

**DOI:** 10.7554/eLife.46561

**Published:** 2019-05-03

**Authors:** Koen B Pouwels, Christopher C Butler, Julie V Robotham

**Affiliations:** 1 Health Economics Research Centre, Nuffield Department of Population HealthUniversity of OxfordOxfordUnited Kingdom; 2 Modelling and Economics Unit, National Infection ServicePublic Health EnglandLondonUnited Kingdom; 3 Nuffield Department of Primary Care Health SciencesUniversity of OxfordOxfordUnited Kingdom; Imperial College LondonUnited Kingdom; Imperial College LondonUnited Kingdom

**Keywords:** bacteria, antibiotic resistance, epidemiology, antimicrobial, antibiotic use, *E. coli*, *S. pyogenes*

## Abstract

We are writing to comment on the recent study by Olesen et al., 2018 on antibiotic use and antibiotic resistance.

## Introduction

Countries are increasingly implementing strategies to reduce antibiotic prescribing in an attempt to decrease antibiotic resistance. It should be possible to achieve relatively large reductions in antibiotic prescribing by focusing on prescribing events for acute and uncomplicated presentations for respiratory tract infections (Smieszek et al., 2018; Pouwels et al., 2018a). However, reducing antibiotic prescriptions in more complex cases, such as in patients receiving repeat prescriptions for chronic recurrent infections, may be more difficult. It is important to know whether focussing on unnecessary prescriptions for infectious syndromes where there is little chance of benefit from antibiotic treatment is sufficient to halt or lower antibiotic resistance levels. Olesen *et al.* recently reported that repeat (intensive) use had a weaker association with resistance than first (extensive) use, suggesting that it may indeed be sufficient to focus on the latter (Olesen et al., 2018).

Their analysis uses cross-sectional state-level data, which makes it difficult to draw conclusions. Causal inference based on such data is difficult because it requires several, often implausible, assumptions (Reichenheim and Coutinho, 2010). A fundamental requirement is that reverse causality should not play a role, i.e. antibiotic resistance should not influence antibiotic prescribing. This assumption is highly unlikely to hold. If there is a high prevalence of resistance against a specific antibiotic, or even perceived high prevalence, doctors are likely to prescribe that antibiotic only to patients that are at low risk of having a resistant infection, e.g. patients who have not been recently exposed to that antibiotic (first-time users). Under these circumstances, doctors are likely to avoid that particular antibiotic in patients who have a high risk of having a resistant infection, e.g. patients recently exposed to that antibiotic (repeat users). In case resistance against a commonly prescribed antibiotic is low, the pre-test probability of resistance will likely be low among all patients including those recently exposed to that antibiotic, making substitution less relevant. Of note, various other factors that correlate with antibiotic resistance, such as comorbidity and age, also influence antibiotic prescribing decisions.

Thus, reverse causality may influence repeat use more than first use, potentially contributing to the observed associations. While the choice of which antibiotic is needed is likely influenced by antibiotic resistance levels, the choice of whether or not to prescribe an antibiotic is to a much smaller extent influenced by antibiotic resistance. Therefore, an analysis focusing on overall antibiotic use is likely less susceptible to reverse causality. We therefore decided to evaluate whether repeat use had a stronger association with resistance when using any antibiotic use instead of one specific antibiotic to create the first-time and repeat use predictors. Here we report the results showing that repeat use has a stronger association with antibiotic resistance than first use when focusing on overall use. We also discuss why both the previous and our analysis should not be interpreted as being causal.

## Results

**Figure 1.**
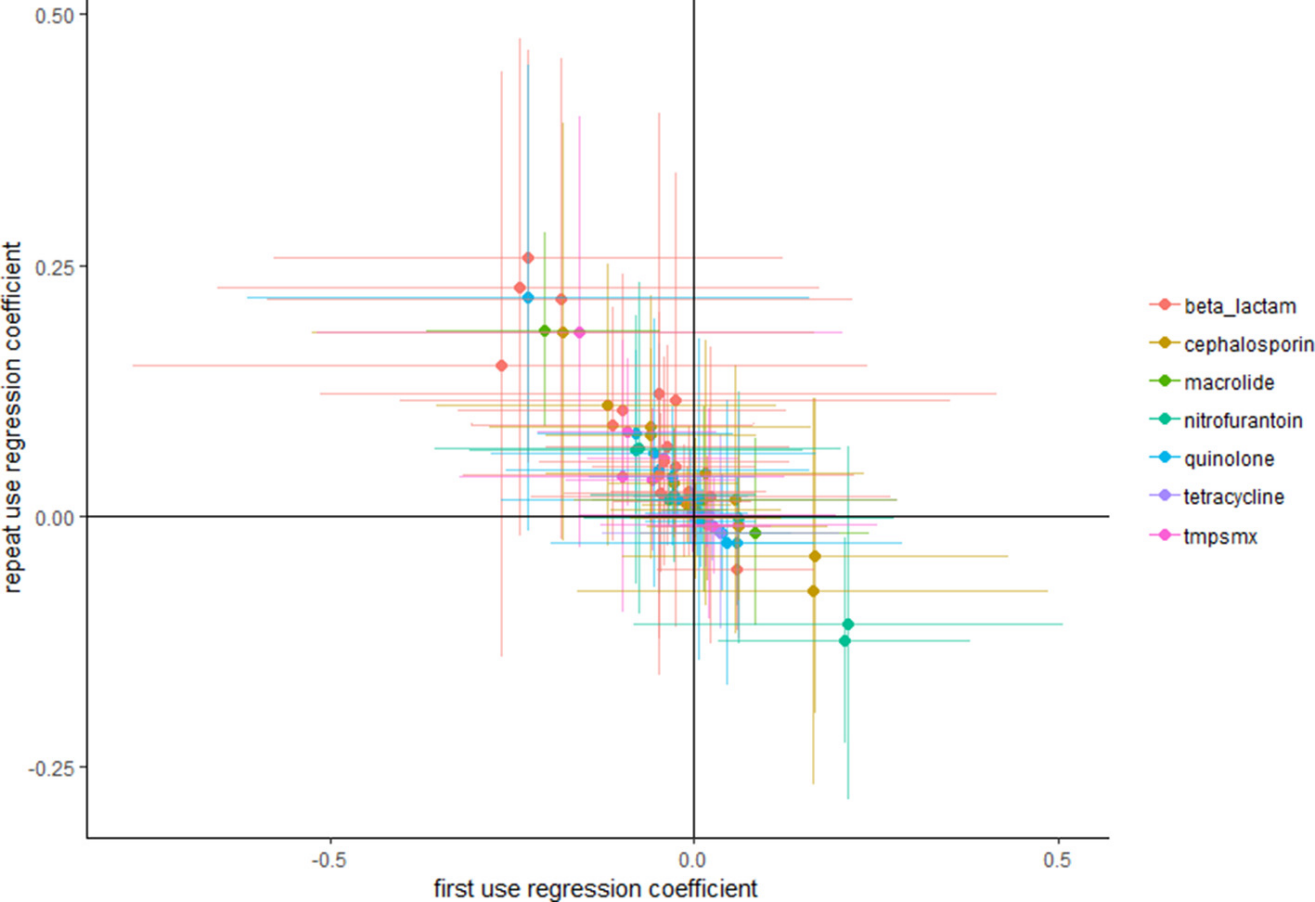
Repeat use tends to be more positively associated with resistance than first use. Each point represents a pathogen–antibiotic combination. The position of the points show the two coefficients from the regressions. The units of the coefficients are proportion resistant per annual claim per 10 people. Error bars show 95% confidence intervals.

When using the data Olesen et al. (2018) used in their main analysis, but focusing on use of any antibiotic, instead of use of particular antibiotics (see Methods): intensive use is more often positively associated with high resistance than extensive use (Figure 1).

By focusing solely on the point estimates one would conclude, if one would assume the associations from Figure 1 are causal, that overall repeat use is more often positively associated with resistance than first use. However, focusing on point estimates only, as Olesen et al. (2018) seem to have done, does not necessarily adequately take into account the relatively large variance. Multicollinearity is known to frequently cause implausible effect estimates and wrong signs of the coefficients. We agree that unbiased estimates can still be obtained despite the presence of multicollinearity: however, this does *not* mean that the point estimate of a specific study should be expected to be close to the 'true' value (Farrar and Glauber, 1967). Deviations from the true value are especially expected when using relatively small datasets, such as those used by Olesen et al. (2018). To evaluate whether reliance on point estimates alone is nevertheless reasonable we evaluated whether similar conclusions would be reached when performing 10,000 bootstrap replications. Supplementary file 1 shows that similar results were obtained when bootstrapping, with only five occasions where the mean of the bootstrap samples had a different sign.

## Discussion

Our results show that when evaluating the association between overall use of antibiotics and antibiotic resistances that repeat use has a stronger association with antibiotic resistance than first use. These results are in the opposite direction of the results of Olesen et al. (2018).

To better understand why these contrasting results may be obtained it is important to consider the relative contributions of forward and reverse causation. Use of one antibiotic may select for or even against resistance to another antibiotic via various mechanisms, such as co-selection, collateral resistance, collateral sensitivity, or by simply killing competing bacteria (Pouwels et al., 2018b; Imamovic and Sommer, 2013; Baym et al., 2016; Kahlmeter and Menday, 2003). Therefore, only considering use of one antibiotic may not be sufficient to obtain the effect of that antibiotic on resistance against that same antibiotic, even in the absence of other biases (Pouwels et al., 2018b). Moreover, resistance against sulphonamides remained high despite drastic reductions in sulphonamide prescribing, largely driven by co-selection via use of other antibiotics (Enne et al., 2001). While 'overall antibiotic use' does partly account for selection of resistance via other antibiotics, it likely includes antibiotics that do not select for antibiotic resistance, thereby potentially attenuating forward causation compared to an analysis with an exposure measure that only includes antibiotics that do select for antibiotic resistance. However, as explained above, overall antibiotic use may be less susceptible to reverse causality because antibiotic resistance has likely a stronger influence on which antibiotic to prescribe than on the decision to prescribe an antibiotic or not. It could be argued that with higher levels of resistance, more unique drugs will be required to cure an infection, making overall use susceptible to reverse causality, however treatment failures due to antibiotic resistance are likely underlying only a small proportion of treatment switches among repeat users in the outpatient setting (Coenen and Goossens, 2014).

Whether the results can be interpreted as the causal effects of first and repeat use of antibiotics on antibiotic resistance levels depends on several assumptions. We want to stress that both our as well as the previous analyses of Olesen et al. (2018) are likely affected by several other biases. Sicker patients require more antibiotics and are likely also be more prone to acquire resistant pathogens independent of antibiotic use in the community. Indeed, a substantial number of studies suggest that comorbidities are associated with increased antibiotic resistance levels, even after accounting for antibiotic use (Chatterjee et al., 2018). Furthermore, while on average a large proportion of antibiotics are being used in an outpatient setting, specific antibiotics may be much more frequently used in the inpatient setting, for which no data is included. For example, in the UK > 40% of cephalosporin prescriptions are among hospital in-patients (ESPAUR writing committee, 2018), questioning the validity of some of the analyses if similar patterns of use were to be observed in the US.

A potentially more fundamental problem is that the distribution of different specimen types across States is unclear. The prevalence of resistance likely differs between different types of specimens. For example, in England approximately 18% of *Escherichia coli* blood samples were resistant to ciprofloxacin, while approximately 11% of urinary *E. coli* samples were resistant to ciprofloxacin in England in 2015 (https://fingertips.phe.org.uk/). Analyses are likely further complicated by variation in the likelihood of sending specific specimens for susceptibility testing. It has previously been found that a substantial part of the variation in (apparent) antibiotic resistance prevalence against trimethoprim among *Klebsiella Pneumoniae* and *Proteus mirabilis* can be explained by variation of the number of urinary samples tested per population (Pouwels et al., 2018b). If patterns of antibiotic use correlate with the likelihood of taking samples, for example because doctors are more likely to send a sample for testing among patients that were already exposed to antibiotics multiple times, associations will suffer from substantial bias.

Given the facts that i) treatment failures and repeat use of antibiotics increase the chances that a clinician will send a sample for susceptibility testing; and ii) there is more repeat than first use of antibiotics (Olesen et al., 2018; Shallcross et al., 2017; Dolk et al., 2018), the majority of specimens are expected to be taken from patients exposed to repeat antibiotics and at higher risk of antibiotic resistance. It remains a question whether variation in resistance among specimens taken from such patients would be more driven by their own intensive use or extensive use of antibiotics by other patients.

In conclusion, given the limitations discussed and the observation that repeat use has a stronger association with resistance than first use when focusing on overall antibiotic use, the conclusion that first-use has a bigger impact on resistance than repeat use of antibiotics is not justified (yet).

## Materials and methods

We used the antibiotic use data and antibiotic resistance data that accompanied the by Olesen et al. Details about the data sources are described in Olesen et al. (2018). We replicated their main analysis with the exception that we focused on overall use of antibiotics instead of specific antibiotic use – antibiotic resistance pairs. For example, instead of evaluating the association between use of first and repeat use of quinolone and quinolone resistance among *E. coli,* we analysed the association between use of first and repeat use of any antibiotic and quinolone resistance among *E. coli*. Multiple linear regressions were performed for the different bug-drug combinations. We compared these results with results obtained when performing 10,000 bootstrap samples for each bug–drug combination. States were sampled with replacement and a linear regression was performed on each sample.

## Data Availability

The data used for this work can be accessed via the paper of Olesen et al (2018): https://elifesciences.org/articles/39435.

## Additional files

Supplementary file 1. Repeat use and first use coefficients from the linear regression using the observed versus bootstrapped data.

Transparent reporting form
