# Supplementary material for: Comment on 'The distribution of antibiotic use and its association with antibiotic resistance'
Source: eLife. 2019 May 3;8:e46561. doi: 10.7554/eLife.46561 (PMC6499535; doi:10.7554/eLife.46561)
Supplement: Supplementary file 1. [file elife-46561-supp1.docx]

**Supplementary File 1. Repeat use and first use coefficients from the linear regression using the observed versus bootstrapped data.**

| **Pathogen** | **Antibiotic resistance** | **Type of use** | **Observed data, β (95% CI)** | **Bootstrapped data,**  **Β (95% CI)** |
| --- | --- | --- | --- | --- |
| *Acinetobacter baumannii* | beta_lactam | First | -0.2280 (-0.5782 to 0.1222) | -0.2266 (-0.5966 to 0.1461) |
|  |  | Repeat | 0.2580 ( 0.0523 to 0.4638) | 0.2565 ( 0.0505 to 0.4874) |
|  | cephalosporin | First | -0.1802 (-0.5258 to 0.1655) | -0.1782 (-0.5702 to 0.2266) |
|  |  | Repeat | 0.1835 (-0.0250 to 0.3920) | 0.1808 (-0.0689 to 0.4160) |
|  | quinolone | First | -0.2281 (-0.6144 to 0.1582) | -0.2248 (-0.5852 to 0.1788) |
|  |  | Repeat | 0.2171 (-0.0145 to 0.4488) | 0.2147 (-0.0183 to 0.4272) |
|  | tmpsmx | First | -0.1577 (-0.5191 to 0.2038) | -0.1589 (-0.4998 to 0.1980) |
|  |  | Repeat | 0.1833 (-0.0313 to 0.3980) | 0.1830 (-0.0314 to 0.4007) |
| *Citrobacter freundii* | beta_lactam | First | -0.2412 (-0.6557 to 0.1732) | -0.2434 (-0.6297 to 0.1429) |
|  |  | Repeat | 0.2280 (-0.0202 to 0.4763) | 0.2288 ( 0.0135 to 0.4422) |
|  | cephalosporin | First | 0.0157 (-0.2036 to 0.2351) | 0.0232 (-0.2047 to 0.2659) |
|  |  | Repeat | 0.0425 (-0.0892 to 0.1741) | 0.0377 (-0.1069 to 0.1630) |
|  | nitrofurantoin | First | -0.0277 (-0.1413 to 0.0859) | -0.0329 (-0.1560 to 0.0662) |
|  |  | Repeat | 0.0205 (-0.0469 to 0.0880) | 0.0234 (-0.0375 to 0.0979) |
|  | quinolone | First | -0.0042 (-0.0823 to 0.0738) | -0.0056 (-0.0762 to 0.0590) |
|  |  | Repeat | 0.0191 (-0.0281 to 0.0663) | 0.0198 (-0.0224 to 0.0680) |
|  | tmpsmx | First | -0.0587 (-0.1756 to 0.0582) | -0.0634 (-0.1924 to 0.0492) |
|  |  | Repeat | 0.0357 (-0.0352 to 0.1065) | 0.0383 (-0.0270 to 0.1130) |
| Coagulase-negative staphylocci (CoNS) | beta_lactam | First | -0.0990 (-0.3244 to 0.1264) | -0.0973 (-0.2760 to 0.0936) |
|  |  | Repeat | 0.1059 (-0.0287 to 0.2405) | 0.1049 (-0.0100 to 0.2223) |
|  | cephalosporin | First | 0.1661 (-0.0999 to 0.4322) | 0.1637 (-0.0547 to 0.3563) |
|  |  | Repeat | -0.0404 (-0.1976 to 0.1168) | -0.0387 (-0.1528 to 0.0888) |
|  | macrolide | First | 0.0130 (-0.1396 to 0.1656) | 0.0165 (-0.1260 to 0.1773) |
|  |  | Repeat | 0.0164 (-0.0759 to 0.1086) | 0.0131 (-0.0886 to 0.0975) |
|  | nitrofurantoin | First | 0.0076 (-0.0221 to 0.0374) | 0.0070 (-0.0204 to 0.0306) |
|  |  | Repeat | -0.0010 (-0.0186 to 0.0166) | -0.0006 (-0.0149 to 0.0161) |
|  | quinolone | **First** | **0.0072 (-0.2655 to 0.2799)** | **-0.0001 (-0.2866 to 0.2747)** |
|  |  | Repeat | 0.0158 (-0.1450 to 0.1765) | 0.0165 (-0.1595 to 0.1776) |
|  | tetracycline | First | 0.0247 (-0.0335 to 0.0829) | 0.0235 (-0.0264 to 0.0799) |
|  |  | Repeat | -0.0103 (-0.0450 to 0.0245) | -0.0101 (-0.0462 to 0.0204) |
|  | tmpsmx | First | 0.0618 (-0.1290 to 0.2527) | 0.0620 (-0.1286 to 0.2677) |
|  |  | Repeat | -0.0085 (-0.1230 to 0.1060) | -0.0103 (-0.1409 to 0.1079) |
| *Enterobacter aerogenes (Klebsiella aerogenes)* | beta_lactam | First | -0.1840 (-0.5871 to 0.2192) | -0.1875 (-0.5318 to 0.1404) |
|  |  | Repeat | 0.2163 (-0.0233 to 0.4559) | 0.2169 ( 0.0298 to 0.4193) |
|  | cephalosporin | First | -0.0605 (-0.2814 to 0.1604) | -0.0582 (-0.2420 to 0.1450) |
|  |  | Repeat | 0.0886 (-0.0428 to 0.2200) | 0.0864 (-0.0322 to 0.1905) |
|  | nitrofurantoin | First | -0.0769 (-0.3562 to 0.2025) | -0.0691 (-0.3109 to 0.1701) |
|  |  | Repeat | 0.0677 (-0.0976 to 0.2330) | 0.0626 (-0.0882 to 0.2002) |
|  | quinolone | First | 0.0035 (-0.0678 to 0.0748) | 0.0046 (-0.0443 to 0.0628) |
|  |  | Repeat | 0.0032 (-0.0395 to 0.0460) | 0.0024 (-0.0351 to 0.0317) |
|  | tmpsmx | **First** | **-0.0019 (-0.0615 to 0.0577)** | **0.0001 (-0.0435 to 0.0560)** |
|  |  | **Repeat** | **0.0009 (-0.0349 to 0.0367)** | **-0.0004 (-0.0370 to 0.0270)** |
| *Enterobacter cloacae* | beta_lactam | First | -0.0266 (-0.4053 to 0.3520) | -0.0193 (-0.3628 to 0.3216) |
|  |  | Repeat | 0.1150 (-0.1119 to 0.3418) | 0.1100 (-0.0896 to 0.3066) |
|  | cephalosporin | First | 0.0569 (-0.1660 to 0.2797) | 0.0620 (-0.1465 to 0.2779) |
|  |  | Repeat | 0.0165 (-0.1170 to 0.1500) | 0.0127 (-0.1188 to 0.1302) |
|  | nitrofurantoin | First | 0.0614 (-0.1521 to 0.2750) | 0.0618 (-0.1971 to 0.2865) |
|  |  | Repeat | -0.0019 (-0.1273 to 0.1234) | -0.0042 (-0.1385 to 0.1425) |
|  | quinolone | First | 0.0585 (-0.0478 to 0.1647) | 0.0593 (-0.0157 to 0.1629) |
|  |  | Repeat | -0.0262 (-0.0899 to 0.0374) | -0.0271 (-0.1007 to 0.0243) |
|  | tmpsmx | First | 0.0145 (-0.0698 to 0.0988) | 0.0156 (-0.0523 to 0.0885) |
|  |  | Repeat | -0.0013 (-0.0518 to 0.0492) | -0.0018 (-0.0452 to 0.0401) |
| *Escherichia coli* | beta_lactam | First | -0.0269 (-0.1407 to 0.0869) | -0.0235 (-0.1342 to 0.1061) |
|  |  | Repeat | 0.0498 (-0.0186 to 0.1183) | 0.0468 (-0.0330 to 0.1194) |
|  | cephalosporin | First | -0.0277 (-0.1168 to 0.0614) | -0.0280 (-0.1017 to 0.0304) |
|  |  | Repeat | 0.0325 (-0.0211 to 0.0861) | 0.0328 (-0.0024 to 0.0792) |
|  | nitrofurantoin | First | 0.0012 (-0.0452 to 0.0476) | 0.0005 (-0.0449 to 0.0456) |
|  |  | Repeat | 0.0061 (-0.0212 to 0.0333) | 0.0061 (-0.0215 to 0.0343) |
|  | quinolone | First | -0.0807 (-0.2157 to 0.0543) | -0.0795 (-0.1751 to 0.0263) |
|  |  | Repeat | 0.0830 ( 0.0018 to 0.1642) | 0.0819 ( 0.0130 to 0.1416) |
|  | tmpsmx | First | -0.0429 (-0.1477 to 0.0618) | -0.0397 (-0.1369 to 0.0759) |
|  |  | Repeat | 0.0580 (-0.0050 to 0.1210) | 0.0560 (-0.0135 to 0.1146) |
| *Enterococcus faecalis* | beta_lactam | First | -0.0080 (-0.1156 to 0.0995) | -0.0071 (-0.1069 to 0.0555) |
|  |  | Repeat | 0.0243 (-0.0402 to 0.0888) | 0.0226 (-0.0294 to 0.1130) |
|  | nitrofurantoin | First | -0.0010 (-0.0372 to 0.0353) | -0.0010 (-0.0360 to 0.0267) |
|  |  | Repeat | 0.0060 (-0.0156 to 0.0276) | 0.0055 (-0.0140 to 0.0341) |
|  | quinolone | First | -0.0495 (-0.2589 to 0.1599) | -0.0510 (-0.2432 to 0.1375) |
|  |  | Repeat | 0.0464 (-0.0779 to 0.1708) | 0.0465 (-0.0679 to 0.1596) |
|  | beta_lactam | First | -0.0408 (-0.2132 to 0.1315) | -0.0422 (-0.2229 to 0.1130) |
|  |  | Repeat | 0.0535 (-0.0507 to 0.1577) | 0.0548 (-0.0428 to 0.1696) |
| *Klebsiella oxytoca* | beta_lactam | First | -0.0486 (-0.3169 to 0.2197) | -0.0453 (-0.3173 to 0.2354) |
|  |  | Repeat | 0.0405 (-0.1227 to 0.2036) | 0.0360 (-0.1371 to 0.1851) |
|  | cephalosporin | First | 0.0600 (-0.0637 to 0.1837) | 0.0520 (-0.0932 to 0.1907) |
|  |  | Repeat | -0.0102 (-0.0854 to 0.0650) | -0.0050 (-0.0845 to 0.0827) |
|  | nitrofurantoin | First | 0.2070 ( 0.0333 to 0.3808) | 0.2076 ( 0.0068 to 0.4176) |
|  |  | Repeat | -0.1251 (-0.2278 to -0.0224) | -0.1267 (-0.2547 to -0.0084) |
|  | quinolone | First | 0.0095 (-0.0358 to 0.0548) | 0.0092 (-0.0352 to 0.0568) |
|  |  | Repeat | -0.0011 (-0.0286 to 0.0264) | -0.0006 (-0.0268 to 0.0265) |
|  | tmpsmx | First | 0.0024 (-0.0487 to 0.0535) | 0.0045 (-0.0474 to 0.0658) |
|  |  | **Repeat** | **0.0009 (-0.0302 to 0.0319)** | **-0.0005 (-0.0345 to 0.0251)** |
| *Klebsiella pneumoniae* | beta_lactam | First | -0.1126 (-0.3068 to 0.0816) | -0.1151 (-0.3394 to 0.1024) |
|  |  | Repeat | 0.0914 (-0.0254 to 0.2081) | 0.0924 (-0.0397 to 0.2208) |
|  | cephalosporin | First | 0.0025 (-0.1141 to 0.1190) | 0.0027 (-0.0909 to 0.1120) |
|  |  | Repeat | 0.0067 (-0.0634 to 0.0768) | 0.0067 (-0.0586 to 0.0627) |
|  | nitrofurantoin | First | -0.0805 (-0.3097 to 0.1488) | -0.0712 (-0.3107 to 0.2082) |
|  |  | Repeat | 0.0661 (-0.0680 to 0.2003) | 0.0591 (-0.1157 to 0.2015) |
|  | quinolone | First | 0.0380 (-0.0582 to 0.1343) | 0.0396 ( 0.0346 to 0.1379) |
|  |  | Repeat | -0.0164 (-0.0743 to 0.0415) | -0.0175 (-0.0777 to 0.0249) |
|  | tmpsmx | First | 0.0260 (-0.0539 to 0.1060) | 0.0286 (-0.0409 to 0.1111) |
|  |  | Repeat | -0.0109 (-0.0589 to 0.0372) | -0.0121 (-0.0613 to 0.0290) |
| *Morganella* | beta_lactam | First | -0.2659 (-0.7716 to 0.2399) | -0.2377 (-0.6801 to 0.2906) |
|  |  | Repeat | 0.1502 (-0.1417 to 0.4421) | 0.1384 (-0.1683 to 0.3955) |
| *Pseudomonas aeruginosa* | beta_lactam | First | -0.0367 (-0.2041 to 0.1306) | -0.0394 (-0.1889 to 0.0917) |
|  |  | Repeat | 0.0697 (-0.0309 to 0.1704) | 0.0705 (-0.0175 to 0.1772) |
|  | cephalosporin | First | -0.0591 (-0.2032 to 0.0851) | -0.0579 (-0.1971 to 0.0758) |
|  |  | Repeat | 0.0799 (-0.0068 to 0.1666) | 0.0781 (-0.0010 to 0.1707) |
|  | quinolone | First | -0.0307 (-0.1455 to 0.0840) | -0.0326 (-0.1274 to 0.0565) |
|  |  | Repeat | 0.0392 (-0.0298 to 0.1082) | 0.0396 (-0.0114 to 0.0956) |
| *Proteus mirabilis* | beta_lactam | First | -0.0153 (-0.1091 to 0.0785) | -0.0194 (-0.1136 to 0.0658) |
|  |  | Repeat | 0.0140 (-0.0422 to 0.0702) | 0.0163 (-0.0319 to 0.0728) |
|  | cephalosporin | First | 0.0167 (-0.0867 to 0.1202) | 0.0109 (-0.0953 to 0.0896) |
|  |  | **Repeat** | **-0.0025 (-0.0645 to 0.0595)** | **0.0011 (-0.0404 to 0.0596)** |
|  | nitrofurantoin | First | 0.2125 (-0.0827 to 0.5077) | 0.2236 (-0.0718 to 0.6081) |
|  |  | Repeat | -0.1073 (-0.2832 to 0.0687) | -0.1144 (-0.3181 to 0.0267) |
|  | quinolone | First | -0.0550 (-0.2782 to 0.1683) | -0.0618 (-0.2863 to 0.1572) |
|  |  | Repeat | 0.0627 (-0.0710 to 0.1965) | 0.0664 (-0.0610 to 0.2000) |
|  | tmpsmx | First | 0.0187 (-0.1577 to 0.1950) | 0.0187 (-0.1531 to 0.1935) |
|  |  | Repeat | 0.0020 (-0.1036 to 0.1076) | 0.0023 (-0.1041 to 0.1058) |
| *Staphylococcus aureus* | beta_lactam | First | 0.0224 (-0.2254 to 0.2701) | 0.0240 (-0.2050 to 0.2680) |
|  |  | Repeat | 0.0199 (-0.1285 to 0.1684) | 0.0163 (-0.1364 to 0.1554) |
|  | cephalosporin | First | 0.1633 (-0.1609 to 0.4875) | 0.1554 (-0.2192 to 0.5109) |
|  |  | Repeat | -0.0752 (-0.2677 to 0.1173) | -0.0736 (-0.2939 to 0.1403) |
|  | macrolide | First | 0.0837 (-0.0738 to 0.2412) | 0.0848 (-0.0656 to 0.2682) |
|  |  | Repeat | -0.0162 (-0.1102 to 0.0778) | -0.0168 (-0.1350 to 0.0726) |
|  | nitrofurantoin | First | -0.0345 (-0.0782 to 0.0093) | -0.0332 (-0.0932 to 0.0212) |
|  |  | Repeat | 0.0161 (-0.0099 to 0.0421) | 0.0149 (-0.0179 to 0.0482) |
|  | quinolone | First | 0.0446 (-0.1974 to 0.2865) | 0.0423 (-0.1730 to 0.2618) |
|  |  | Repeat | -0.0276 (-0.1697 to 0.1146) | -0.0285 (-0.1637 to 0.0845) |
|  | tetracycline | First | -0.0039 (-0.0431 to 0.0353) | -0.0051 (-0.0402 to 0.0293) |
|  |  | Repeat | 0.0050 (-0.0184 to 0.0285) | 0.0057 (-0.0149 to 0.0266) |
|  | tmpsmx | First | 0.0178 (-0.0193 to 0.0549) | 0.0198 (-0.0106 to 0.0573) |
|  |  | Repeat | -0.0088 (-0.0311 to 0.0134) | -0.0097 (-0.0307 to 0.0069) |
| *Streptococcus pneumoniae* | beta_lactam | First | -0.0474 (-0.1786 to 0.0839) | -0.0451 (-0.1680 to 0.0893) |
|  |  | Repeat | 0.0230 (-0.0560 to 0.1019) | 0.0225 (-0.0592 to 0.1009) |
|  | cephalosporin | First | -0.0100 (-0.0721 to 0.0521) | -0.0081 (-0.0810 to 0.0724) |
|  |  | Repeat | 0.0113 (-0.0261 to 0.0488) | 0.0107 (-0.0352 to 0.0561) |
|  | macrolide | First | -0.2064 (-0.3671 to -0.0457) | -0.2120 (-0.4120 to -0.0391) |
|  |  | Repeat | 0.1853 ( 0.0886 to 0.2820) | 0.1885 ( 0.0812 to 0.3143) |
|  | quinolone | First | -0.0242 (-0.0567 to 0.0083) | -0.0252 (-0.0531 to -0.0012) |
|  |  | Repeat | 0.0164 (-0.0032 to 0.0360) | 0.0169 ( 0.0036 to 0.0325) |
|  | tetracycline | First | 0.0369 (-0.1272 to 0.2010) | 0.0443 (-0.0954 to 0.2201) |
|  |  | Repeat | -0.0166 (-0.1135 to 0.0804) | -0.0212 (-0.1379 to 0.0662) |
|  | tmpsmx | First | -0.0919 (-0.2148 to 0.0309) | -0.0862 (-0.2230 to 0.0571) |
|  |  | Repeat | 0.0834 ( 0.0095 to 0.1574) | 0.0799 (-0.0069 to 0.1668) |
| *Serratia* | beta_lactam | First | -0.0480 (-0.5132 to 0.4171) | -0.0446 (-0.5470 to 0.4643) |
|  |  | Repeat | 0.1216 (-0.1588 to 0.4020) | 0.1170 (-0.1691 to 0.3924) |
|  | cephalosporin | First | -0.1198 (-0.3537 to 0.1140) | -0.1774 (-0.5711 to 0.2295) |
|  |  | Repeat | 0.1103 (-0.0309 to 0.2515) | 0.1809 (-0.0687 to 0.4156) |
|  | quinolone | First | 0.0092 (-0.0668 to 0.0852) | 0.0086 (-0.0557 to 0.0726) |
|  |  | Repeat | -0.0055 (-0.0513 to 0.0404) | -0.0052 (-0.0448 to 0.0325) |
|  | tmpsmx | First | -0.0987 (-0.3223 to 0.1249) | -0.1040 (-0.3240 to 0.0072) |
|  |  | Repeat | 0.0397 (-0.0958 to 0.1752) | 0.0420 (-0.0046 to 0.1336) |
| *Stenotrophomonas* | tmpsmx | First | 0.0583 (-0.0498 to 0.1664) | 0.0586 (-0.0510 to 0.1856) |
|  |  | Repeat | -0.0534 (-0.1148 to 0.0080) | -0.0534 (-0.1264 to 0.0131) |

Cases where the sign of the coefficients differ are highlighted in bold.
